# A road less travelled: Undergraduate midwifery students’ experiences of a decentralised clinical training platform

**DOI:** 10.4102/hsag.v25i0.1473

**Published:** 2020-10-08

**Authors:** Olivia B. Baloyi, Gugu G. Mchunu, Charlene Williams, Mary-Ann Jarvis

**Affiliations:** 1 Department of Nursing, Faculty of Health Sciences, KwaZulu-Natal University, Durban, South Africa

**Keywords:** decentralised clinical training platforms, midwifery, nurses, rural hospitals, undergraduate student

## Abstract

**Background:**

In South Africa, the critical skill base shortage of healthcare workers, the underperforming global health indicators and the planned roll out of the National Health Insurance have burdened South African higher education authorities to rapidly expand nursing student enrolments. The expansion in student numbers has placed increased demands on overstretched educational institutions, and students are confronted with challenges of congestion in classrooms and clinical facilities, while lecturers encounter difficulties in the process of clinical allocation. A solution is to utilise decentralised clinical training platforms (DCTPs) and allocate students in rural hospitals.

**Aim:**

To explore and describe undergraduate midwifery students’ reflections of their DCTP experiences, in order to inform future practice of decentralisation in student training.

**Setting:**

The study was conducted in the nursing discipline of an urban-based university in KwaZulu-Natal, South Africa, involving undergraduate midwifery students. The university had commenced a programme of allocating students to decentralised clinical sites.

**Method:**

Elo and Kyngäs’ content analysis was used to analyse the experiences of DCTP by undergraduate midwifery students (*n* = 14) as expressed in a focus group (*n* = 11) and three individual interviews (*n* = 3).

**Results:**

The following four categories emerged: Recognition as a team member, engaging support, win–win platform and juxtaposed challenges.

**Conclusion:**

In the presence of support and teamwork, rural settings can develop undergraduate student midwives, not only in the areas of midwifery competency but also in their personal capacity, and strengthen the responsiveness, preparedness and relevance of midwifery graduates.

## Background

Africa strives to honour its commitment to Universal Health Coverage (UHC), but a healthcare worker shortage threatens this goal (WHO 2016a). World Health Organization (WHO) (2016a) has projected that by 2030 Africa will experience a shortage of 4.2 million healthcare workers, of whom 2.8 million will be nurses and midwives. In South Africa, the skill-based shortage is of concern in a healthcare context characterised by global health indicators such as mounting maternal mortality and morbidity rates (Saving Mothers 2017; WHO 2016b), increased under-five mortality rates (World Bank 2019) and a life expectancy lower than the global average (Worlometer 2020), further complicated by social determinants of health such as poverty (Ataguba, Day & McIntyre 2015). These critical shortages, the changing healthcare landscape and the planned roll out of the National Health Insurance have burdened South African higher educational authorities to rapidly expand nursing student enrolments (de Villiers et al. 2017; Fakude et al. 2014). Student enrolments have shown an expansion in numbers and increased demands on educational institutions (de Villiers et al. 2017; Fakude et al. 2014). Students enrolling in these already overstretched institutions are confronted with numerous challenges that include, but are not limited to, congestion in classrooms (Fakude et al. 2014) and clinical facilities, while lecturers encounter difficulties in the process of clinical allocation (de Villiers et al. 2017).

WHO (2013) and local associations, such as the South African Association of Health Educationalists (SAAHE), have deliberated on how to meet these clinical placement challenges (Gaede 2018). An option explored by various South African universities was to increase the clinical platform through the decentralisation of training (Gaede 2018; WHO 2013). Decentralisation of training is also referred to as community-based training framed within a primary healthcare model (National Department of Health South Africa 2015), or community-based medical education (Kelly, Walters & Rosenthal 2014), peripheral learning, bush learning and distributed training (De Villiers et al. 2017; Mlambo et al. 2018). A decentralised clinical training platform (DCTP), such as the Stellenbosch University Collaborative Capacity Enhancement through Engagement with Districts (SUCCEED), shifts training from traditional, urban-based healthcare settings, localised around the academic structures, to rural communities (De Villiers et al. 2017; van Staden 2019). The outcomes of DCTPs have shown healthcare system value, such as increased graduate output (Gaede 2018), addressing the current urban and rural personnel maldistribution (de Villiers et al. 2017). In addition, a DCTP has relevance for graduates themselves in the opportunity to increase their sense of significance in a multi-professional context (de Villiers et al. 2017), in exposure to under-resourced settings to develop an awareness of the role of the social determinants of health in midwifery (National Department of Health South Africa 2015), providing a reflective opportunity for future possible practice challenges (Kelly et al. 2014). However, in the literature, the positive outcomes centre on medical students’ education as the predominant recipients of DCTPs, with an under-explored opportunity for nurses and midwives, particularly within the South African context.

The selected university’s nursing discipline faced the challenge of limited clinical sites for the undergraduate midwifery students. The nursing discipline aligned itself with the WHO (2010) recommendations to increase the retention of health workers in rural areas, reasoning that graduates might consider such work settings, thereby addressing the current urban and rural personnel maldistribution. Simultaneously, the discipline realised the value of DTCPs to address the challenge of student saturation in urban sites. In addition, DCTPs complemented the discipline’s community-based approach to education. Because of the aforementioned rationalisation, the discipline decentralised clinical allocation, placing the students at rural-based hospitals, thus creating a need for this study to understand their experiences.

## Aim

To explore and describe undergraduate midwifery students’ reflections of their DCTP experiences, in order to inform future practice of decentralisation in student training.

## Research method and design

### Study approach and design

A qualitative content analysis, which was exploratory and descriptive in nature (Grove, Gray & Burns 2015), gained an in-depth insight into the undergraduate midwifery students’ experiences of DCTP. A dearth of literature in this area deemed the approach appropriate (Creswell 2013).

### Study setting

The study was conducted in the nursing discipline of an urban-based university in KwaZulu-Natal, South Africa. At the study setting, the undergraduate nursing qualification is a 4-year degree programme, leading to registration with the South African Nursing Council (SANC) as a nurse (general, community and psychiatry) and midwife. The targeting of rural areas in the recruitment of undergraduate students is to increase the possibility of their return as graduates, in line with the WHO (2010) recommendations to increase the access of skilled healthcare workers to remote areas. Midwifery is a clinical module, completed in the 4th year of study, with mandatory 1000 hours for clinical practicum, as stipulated by SANC regulations (No. R425 1988). Midwifery students were placed at traditional sites in the same city as the university (urban-based hospitals, primary healthcare clinics and community healthcare centres) and for the first time, in 2019, at non-traditional clinical sites (decentralised clinical sites) to develop the learners’ clinical competencies. The midwifery students (*n* = 35) were allocated to DCTP sites for 3 consecutive weeks out of the 17 weeks of clinical placement. The DCTP sites were set in rural areas with limited access to resources, and up-referral involved the mother or the pregnant woman and/or the neonate travelling long distances. Midwives are the backbone of these clinical sites, which have minimal presence of specialist obstetricians. The patients are predominantly isiZulu speaking and come from lower resourced settings.

### Study participants

The target population was all 4th-year undergraduate midwifery students (*n* = 35) studying a Bachelor’s degree in Nursing, in the year 2019, who had participated in the DCTP, and consented to be included in the study.

### Sampling and sample size

Purposive sampling targeted the undergraduate midwifery student group, as they were the first cohort to participate in the DCTP. All 35 students received an invitation to participate in the study, and 14 consented.

### Data collection

The researchers collected the data in 2019 in the university setting, following the students’ exposure to the DCTP sites. Eleven students (P1-11) volunteered to be part of a focus group discussion (FGD), and following this, three students agreed to participate in individual interviews. Data collection continued until no new information was forthcoming, based on the completeness of all categories and sub-categories. Achieving data saturation was through a FGD and three individual in-depth interviews (IND 1–3). The FDG included 11 participants, followed by post-analysis by the three individual interviews. The FGD and interviews were conducted in English, as the medium of instruction in the university. One open-ended question was posed for both the FGD and individual interview: ‘As undergraduate midwifery students, you have all had exposure to the DCTP, what can you share with the group regarding your experiences of the programme?’ Probing questions elicited more information on the phenomenon under study. Rich discussion resulted in the focus group lasting an hour and 16 min, and concluded with no new emerging information, confirmed through the individual interviews, which lasted between 30 and 35 min.

### Ethical considerations

The ethics committee of the university provided ethical approval (HSSREC/00000914/2019) and in addition, the university registrar granted gatekeeper’s permission. All ethical principles were observed when obtaining written consent and during data collection. The provision of pseudonyms, and reiterating the importance of shared information remaining within the data collection setting ensured confidentiality, whilst that of the data was maintained through it being confined to storage in one password locked computer. The FGD and interviews were conducted in private areas. All four researchers acknowledged their dual role as both faculty members and researchers at the study setting, therefore, ensuring no power imbalances during the research process, as described by Kamberelis and Dimitriadis (2005). The participants received assurance that their participation or non-participation in the study would have no impact on their academic progress.

### Data analysis

The FGD and individual interviews were audio-recorded, transcribed verbatim and independently coded by two of the researchers who were not involved in the data collection. The three-phased content analysis (organising, abstraction and reporting) method, described by Elo and Kyngäs’ (2008), guided the coding process in order to explore the DCTP experiences of the participants.

### Trustworthiness

The four criteria employed to validate rigour were credibility, dependability, confirmability and transferability (Lincoln & Guba 1985). Member checking of transcripts with the study participants, citing the participants’ own real words and routine meetings with the co-researchers maximised the trustworthiness of the study findings.

## Results

From the exploration and description of the experiences of the undergraduate midwifery students who had participated in the DCTP, four categories and related sub-categories emerged. Table 1 outlines the accounts of the participants, and presents illustrative quotations from the transcribed data as discussed in the FGD and individual interviews.

**TABLE 1:** Summary of results.

Category	Sub-categories
Category 1: Recognition as a team member	Sub-category 1A: Improved level of confidence as a midwife Sub-category 1B: Negotiating power
Category 2: Engaging support 2a. Peer support	Sub-category 2A: Relationship development
2b. Institutional support	Sub-category 2B: Pre-allocation preparedness
2c. Professional support	Sub-category 2C: Non-competitive support
Category 3: Win–win platform	Sub-category 3A: Broadening vision of outcome
Category 4: Juxtaposed challenges	Sub-category 4A: Limited time Sub-category 4B: Resource limitations

### Category 1: Recognition as a team member

It was important for the participants to have recognition as team members with skill bases in midwifery. In these reflections, two sub-categories emerged.

#### Sub-category 1A: Improved level of confidence as a midwife

The participants’ improved level of confidence contributed to an increase in self-esteem, allowing for a development in their midwifery competency. The following excerpts depict participants’ own words:

‘… For once in my training life I have developed a lot of confidence in what I’m doing as a student midwife, thanks to DCTP for such a wonderful experience.’ (FGD:P7)

‘… for me I have to say my self-esteem improved a lot … like I feel more confident when working with maternity patients.’ (FGD:P6)

‘… DCTP gave me a wonderful experience … I’m loving midwifery and I feel more comfortable and confident as a midwifery student, if given a choice during community service I will choose midwifery.’ (IND:P1)

#### Sub-category 1B: Negotiating power

The participants’ recognition as team members gave them a sense of having power to negotiate midwifery practice plans. The participants explained their negotiating power as follows:

‘… [*T*]he staff here definitely appreciate us as team members (FGD:P1) … We can negotiate patient care plans with other professionals.’ (FGD:P3)

‘… for example on one occasion I had a patient who was not progressing well I informed the doctor, and the two of us negotiated further care, we both agreed on a caesarean section.’ (FGD:P9)

‘… exactly it is that type of power negotiation which makes DCTP different from urban hospitals.’ (FGD:P8)

‘… multi-disciplinary approach at DCTP involved us as students which is usually not a common practice in urban hospitals.’ (IND:P2)

### Category 2: Engaging support

The data sources noted that support in different forms enabled a positive experience of their participation in the DCTP. Support was evident in three forms.

#### Category 2a: Peer support

As peers, the participants found support through their interactions with each other.

**Sub-category 2A: Relationship development:** The DCTP resulted in the participants living together in a communal setting, which exposed them to cross-cultural learning and more personal aspects about each other, which developed their relationships further. The extracts below demonstrate that cross-cultural exposure offered peer support:

‘At DCTP we stay together in a commune … it is through this communal setting that our relationships developed outside classroom and clinical setting … .’ (FGD:P11)

‘… like there is a lot that we do together like sitting together when we cook.’ (FGD:P4)

‘… as non-South African I can now cook phuthu.’ (IND:P2)

Furthermore, the unfamiliarity of the rural geographical location and the healthcare setting resulted in the students drawing on each other’s experiences to develop a sense of solidarity and support:

‘DCTP sites were unfamiliar to most of us … for me it was the first time going to XXX let alone working in a rural hospital … this different setting developed in us as students a lot of solidarity where we learnt to support each other by doing almost everything together.’ (FGD:P7)

‘… like we have planned days … Saturdays were the funniest we used to go town together in a taxi do our groceries … I must say the unity was amazing I know my colleagues much better socially.’ (IND:P1)

#### Category 2b: Institutional support

During the FGD, the participants explained how they derived support from the university and its structures.

**Sub-category 2B: Pre-allocation preparedness:** It emerged that as part of the preparedness for the DCTP, the block system, which aligned classroom teaching and clinical placement, equipped the midwifery students with the necessary knowledge to allow for the transference of theory into practice. The excerpts below show evidence of this occurrence:

‘[*I*] have to commend the university for the block system, it was wonderful, it prepared us well for clinical exposure, we learnt theory first.’ (FGD:P2)

‘… which we then applied in the clinical setting.’ (IND:P3)

‘If I get stuck during patient care I could quickly reflect on what I learnt in class and apply it.’ (IND:P2)

‘On this particular day I had a patient with PIH it was so wonderful to see the theory that I learnt before clinical exposure evolving on that patient.’ (IND:P2)

#### Category 2c: Professional support

A third form of support was derived from the professional staff working at the DCTP sites.

**Sub-category 2C: Non-competitive support:** The participants were outspoken about the rural nature of the setting preventing overcrowding of students in the clinical facilities. The smaller number of students in the DCTP sites placed a lesser demand on the registered midwives to divide their attention among students, which allowed for non-competitive support and exposure to professionals willing to teach:

‘Unlike in the urban based hospitals where we always compete with government students, in rural settings midwives are more willing to teach us.’ (FGD:P3)

‘… because we are not many.’ (FGD:P1)

‘… they give undivided attention making sure that all our learning needs are met.’ (FGD:P6)

‘… the special attention I got from DCTP was commendable … midwives would come and avail themselves to teach.’ (IND:P2)

#### Category 3: Win–win platform

It emerged from the data that there were dual benefits to the individual students and the healthcare system through the placement of the students in the DCTP. It was a win–win platform.

**Sub-category 3A: Broadening vision of outcome:** The participants did not have tunnel vision regarding the DCTP’s value, but were able to broaden their vision to recognise the benefits to the healthcare system. The participants expressed the value of the DCTP in terms of individual outcomes, involving their experience of their personal development, and that of their own midwifery clinical skills, and in addition, they identified the outcome to the healthcare system.

The excerpts below show the broadening vision of the DCTP outcome as seen by the participants:

‘The fact that we are learning so much and practicing independently at DCTP will not only benefit us in meeting our outcomes as student midwives.’ (FGD:P10)

‘… but the healthcare system will also gain itself competent midwives.’ (FGD:P7)

‘… we will be able to function independently as competent midwives during our community service, I think the health department will benefit a lot from us.’ (FGD:P5)

#### Category 4: Juxtaposed challenges

Despite the above positive aspects of team membership and support, the DCTP was not without perceived challenges. As much as the students wanted to stay at the DCTP sites for longer, resource limitations were challenging, as shown in the two sub-categories below.

**Sub-category 4A: Limited time:** The rotational nature of the DCTP allocation placed students at rural sites for three weeks, but the students felt this was too short a period. The students would have preferred a longer period of allocation:

‘… [*T*]he placement period is very short, when we start relaxing, starting to get familiar with the environment and enjoy working and learning the three weeks is over and is almost time to rotate back to XXX.’ (FGD:P8)

‘… what is the possibility of a much longer placement maybe six weeks or more.’ (FGD:P5)

‘… I could have loved to stay a bit longer or at least go back.’ (IND:P3)

**Sub-category 4B: Resource limitations:** The participants found that the rural settings brought with them challenges in relation to both their functioning in the midwifery setting and their role as a student. Resources were limited for academic office support structures, such as photocopying, printing and access to limited Wi-Fi. The clinical setting posed technological challenges in the use of less sophisticated equipment compared to the urban-based hospitals. The participants evidenced these challenges in the extracts below:

‘Eish as much we liked DCTP … erhh resources were a problem unlike here in Durban, there was limited Wi-Fi.’ (FGD:P2)

‘… printing and photocopying was a no no, we had to go to town to print one copy for fifteen rands.’ (IND:P1)

‘… we not used to the equipment we use here … like in urban hospitals we use advanced CTG machines to monitor the mother and her fetus.’ (FGD:P3)

‘… here we use mainly fetoscopes for the same purpose.’ (FGD:P1)

‘… which is kind of a challenge because we not used to that it …’ (FGD:P7)

‘… I could not hear the fetal heart rate through fetoscope.’ (IND:P2)

## Discussion

The participants’ discussion gave little focus to DCTP’s greater benefits, but provided four primary findings for its short-term outcomes.

The first finding highlights the value of midwifery students’ recognition of teamwork. The DCTP sites were unlike the urban-based clinical settings that are often described as overcrowded (De Villiers et al. 2014) and result in students competing for the facilitator’s attention and knowledge (Malwela, Maputle & Lebese 2016; Rikhotso, Williams & De Wet 2014). As seen in this study, the rural settings with smaller number of students appeared to lessen the power discourses, provided for teamwork, and access to clinical learning opportunities (Ahmadi et al. 2018). It is through the DCTP, as in other studies (Talib et al. 2017), that the participants were afforded an opportunity to work with other professionals, for example medical doctors, medical students, obstetricians, midwives and advanced midwives. It is in such settings framed in co-operative approaches of inter-professional collaboration that students are able to improve their self-confidence (Connolly, Sweet, & Campbell 2014; Hosny et al. 2013; Mlambo et al. 2018), and develop their clinical competence (Mlambo et al. 2018), embarking on decision-making as an accountable professional within a safe zone (Mishra 2013). Connolly and colleagues (2014) corroborate with the findings of this study that in settings where teamwork is acknowledged and where members, including the student, hold equal value, a sense of belonging is promoted within the student. The learning opportunities offered in decentralised rural settings are in contrast to urban-based hospitals where students’ voices can become obscured, and, as a result, they can feel powerless (Baraz, Memarian & Vanaki 2015).

The ability to develop self-confidence that allows the student to move from the zone of proximal development, as theorised by Vygotsky (Mishra 2013), tapered to a less dependent and more accountable professional space requires supportive relationships (Jarvis & Baloyi 2020), and brings to the fore the second finding of this study.

The participants were able to recognise that support may come from different structures and social networks. In addition to professional support, as discussed earlier, peers offered a valuable source of support to each other with meaningful social connections in their sense of togetherness. Bonding capital with reciprocity is typical and strong in homogenous groups (Putnam 2000). Mapukata et al. (2017) describe the value of placing students together in a site, and its relevance towards their social learning and development of cultural competence. Whilst this study did not explore the link between culture, environmental background and perceptions, there was evidence that cross-cultural exposure offered peer support amongst the group members.

As much as the students drew on each other for their strength and identified the value of peer support in relationship development, they referred to the importance of institutional support and the preparation that was necessary before their placement at DCTP sites. The cumulative value of support and teamwork contributed to a win–win platform, where students were able to identify the value to them as graduates, as well as extending their vision to identify the healthcare system outcomes. The dual benefit collaborates with the findings by Talib et al. (2017). In Talib et al.’s (2017) study, the medical students suggested that because of their involvement in the DCTP, they became more competent, with a consequential improvement in health indicators, and one area specifically described a decrease in maternal mortality. It is of note that despite the positive short-term outcomes of the DCTP and the broadened vision to the healthcare system, the students failed to give mention to the social determinants of health and the differing social presentations in rural and urban settings.

The last finding centred on the system-related challenges experienced by the undergraduate midwives, which appeared juxtaposed in that as much as the students felt the time was too short, they found the rural nature of the settings challenging. Various authors who describe the DCTP with medical students highlight that 4 to 6 weeks is a preferable period for allocation, unlike the 3-week placement plan in this study (Couper et al. 2009; Doherty & Couper 2016; Kapanda et al. 2018). It takes students time to acclimatise to the rural setting, for example customising themselves to the differences in equipment compared to urban settings (Kapanda et al. 2018).

In addition, the limitation in resources, such as access to Wi-Fi and photocopying facilities, to compliment 21st century education was challenging. Progressive education requires students to be self-directing in their quest for knowledge and consequently the development of higher order thinking skills (Baloyi and Mtshali 2018a), and often times requiring internet access, which can be problematic in rural settings (Doherty & Couper 2016). South Africa’s Network Readiness Index has improved to 65th position out of 139 countries, and 4G connectivity is accessible to about 75% of the population; however, despite plans to connect all South Africans to Internet by 2020, data remain expensive (Naidoo 2017). It is significant to note that despite the evidence provided by WHO (2010) of undergraduate exposure to rural settings as a retention measure, some authors differ and have highlighted that students’ exposure to challenges in rural settings can deter them from selecting to work in such settings when qualified (Talib et al. 2017).

## Limitations

The methodology only allowed a small sample of students to participate in the study.

## Recommendations

A comparative study is recommended involving all health science students from the study university who attended DCTP sites in order to determine their experiences and the curriculum benefits. A study comparing the perceptions of the students at the beginning and at the end of the DCTP placement could be beneficial to influence curriculum changes.

The study findings highlight the importance of pre-placement preparation of students for DCTP, to introduce students to the use of less technologically driven apparatus such as a fetoscope and the differing social determinants of health/illness.

Higher education institutions that adopt a DCTP should allocate students for a period greater than 3 weeks.

## Conclusion

A DCTP offers strong possibilities to the challenges faced by nursing disciplines of higher education institutions, when placing undergraduate student midwives in urban-based hospitals over-burdened with students. In the presence of support and teamwork, rural settings can develop undergraduate student midwives, not only in the areas of midwifery competency but also in their personal capacity. The study findings provide valuable information to nurse educators’ planning to utilise a DCTP as a means to expand clinical training opportunities and contribute towards the equity of human resources for healthcare delivery. An opportunity exists through this platform to strengthen the responsiveness, preparedness and relevance of midwifery graduates who are capable of contributing to UHC and the roll out of the National Health Insurance in lesser resourced rural settings.
